# Transcriptomic Plasticity of Human Alveolar Macrophages Revealed by Single-Cell RNA Sequencing Following Drug Exposure: Implications for Therapeutic Development

**DOI:** 10.3390/ijms26094439

**Published:** 2025-05-07

**Authors:** Penny L. Groves, Levi Hockey, Brendan J. O’Sullivan, Lai-Ying Zhang, Zherui Xiong, Quan H. Nguyen, Maxine E. Tan, Viviana P. Lutzky, Rohan A. Davis, Daniel C. Chambers, Simon H. Apte

**Affiliations:** 1 Queensland Lung Transplant Service, The Prince Charles Hospital, Chermside, QLD 4032, Australia; penelope.groves@health.qld.gov.au (P.L.G.); brendan.osullivan2@health.qld.gov.au (B.J.O.); lai-ying.zhang@health.qld.gov.au (L.-Y.Z.); viviana.lutzky@health.qld.gov.au (V.P.L.); daniel.chambers@health.qld.gov.au (D.C.C.); 2 Faculty of Health, Medicine and Behavioural Sciences, The University of Queensland, St. Lucia, QLD 4072, Australia; l.hockey@uq.edu.au; 3 QIMR Berghofer Medical Research Institute, Herston, QLD 4006, Australia; zherui.xiong@qimrb.edu.au (Z.X.); quan.nguyen@qimrb.edu.au (Q.H.N.); 4 Institute for Molecular Bioscience, The University of Queensland, St. Lucia, QLD 4072, Australia; 5 School of Environment and Science, Griffith University, Nathan, QLD 4111, Australia; r.davis@griffith.edu.au; 6 NatureBank, Institute for Biomedicine and Glycomics, Griffith University, Nathan, QLD 4111, Australia

**Keywords:** pulmonary fibrosis, lung airway macrophages, transcriptional plasticity, drug discovery, natural products, phytochemistry, profibrotic macrophages, pioglitazone, endiandrin A, trametinib, nintedanib, LPS

## Abstract

Alveolar macrophages (AM) must perform three seemingly opposing roles including homeostasis, driving inflammation, and facilitating tissue repair. Whilst there is now consensus (supported by a large body of human single cell RNA sequencing (scRNA-seq) data) that the cell subsets that perform these tasks can readily be found based on their transcriptome, their ontogeny has remained unclear. Moreover, there is agreement that in all types of pulmonary fibrosis (PF) there is an expanded population of profibrotic AM that may aberrantly drive PF. From a therapeutic viewpoint, there is great appeal in the notion that the transcriptional program in different AM subsets is not fixed but remains plastic and amenable to pharmacological reprogramming. Accordingly, this study addresses this question by performing scRNA-seq on human AM following treatment with drugs or perturbagens including pioglitazone, trametinib, nintedanib, lipopolysaccharide and the natural compound endiandrin A. Each treatment induced a unique global transcriptional change, driving the cells towards distinct subsets, further supported by trajectory analysis, confirming a high level of plasticity. Confirmatory experiments using qPCR demonstrated that single exposure to a compound induced a relatively stable transcriptome, whereas serial exposure to a different compound allowed the cells to be reprogrammed yet again to a different phenotype. These findings add new insight into the biology of AM and support the development of novel therapies to treat PF.

## 1. Introduction

Alveolar macrophages (AM) are a unique population of cells that reside in an extra-epithelial location in pulmonary airspaces, in particular the unciliated alveolus, bathed in surfactant. They are often considered as a simple innate immune cell whose main role is to clear the airways of pathogens and foreign particles by phagocytosis, but this is only a small part of their repertoire of diverse capabilities. In humans they number in the billions and play a critical role in maintaining pulmonary homeostasis and responding to diverse stimuli, including pathogens, environmental insults, and therapeutic interventions.

One major and ongoing role of AM is to participate in lung surfactant recycling and homeostasis (reviewed in [1]). It is estimated that adult humans have a surfactant pool of approximately 70 mL [2] that is renewed by a mix of degradation and recycling every 4 h [3], with AM removing 20% of the pool for degradation. Pulmonary surfactant is a mix of complex lipids and proteins that need to be catabolised by AM, and dysregulation of the recycling process due to genetic or idiopathic causes can lead rapidly to pulmonary alveolar proteinosis or acute respiratory distress syndrome (ARDS) [1]. Moreover, the surfactant itself is liable to damage by free radicals and yet another role for AM is the removal of oxidised proteins [4]. It is not surprising therefore that the transcriptome of homeostatic macrophages is dominated by genes related to lipid processing.

Apart from their critical homeostatic roles, AM must be able to dynamically respond to pathogens by sensing, phagocytosing, and driving immune activation by their classical pro-inflammatory functions. Beyond simply driving chemotaxis of innate and adaptive immune cells into the lung airspace, they also participate in antigen processing and presentation and assist adaptive and memory responses (reviewed in [5]).

The lung is very susceptible to immune-response-induced collateral tissue damage, or to other environmental insults including allergens and pollution (reviewed in [6]). The natural repair process following tissue damage is fibrosis and then tissue restoration and AM must dynamically respond to these challenges as well [7]. Aberrant control of the fibrotic processes can lead to pulmonary fibrosis and whilst some factors that drive lung fibrosis are known such as environmental exposure (e.g., silicosis), mutations in genes that maintain telomere length, or viral infection (e.g., post COVID-19 lung fibrosis [8]), for the majority of cases the disease is idiopathic. Idiopathic pulmonary fibrosis (IPF) has an increasing prevalence, a median survival post-diagnosis of between 2–5 years and the only two approved medications (nintedanib and pirfenidone) can at-best slow the progression of the disease, leaving lung transplantation as the only treatment option for end-stage disease.

Based on a recent surge in scRNA-seq studies in human lung, there is now consensus among many research groups that an aberrant population of pro-fibrotic macrophages is driving lung fibrosis in diseases including IPF [9,10,11,12,13], chronic obstructive pulmonary disease (COPD) [9], and post COVID-19 lung fibrosis [14,15]. These pro-fibrotic macrophages appear to share similar transcriptional profiles regardless of the disease and the idea that these macrophages could be reprogrammed and halt the fibrotic process has great appeal and is the focus of considerable research [13,16,17,18,19].

In a previous study, we developed a medium throughput assay utilising human AM to detect compounds with the ability to reprogram a small subset of AM genes that represent either pro-fibrotic or homeostatic subsets [20]. That assay is highly sensitive, but because our assessment was made using qPCR of the entire cell population, it was not clear if the outcomes we observed were the results of a global plasticity, or simply an up or down regulation of specific genes within fixed subsets.

To address this question, in the work presented here, we performed scRNA-seq on human lung macrophages following treatment with single compounds and show that the transcriptional changes are at a global level; and, in a secondary set of experiments show by qPCR that gene expression profiles can first be driven in one direction and then driven toward a different profile by serial exposure to different compounds.

## 2. Results

### 2.1. Human Lung Macrophage Transcriptomics Are Globally Plastic and Amenable to Pharmacological Modulation

Human AM from our established biobank of cryopreserved cells, recovered from patients undergoing whole lung lavage at our hospital as a treatment for silicosis [21], were cultured in vitro using our established protocol ([20] and materials and methods). Briefly, cryopreserved macrophages were thawed and stabilised in culture media overnight, non-adherent cells were removed and the media renewed with inclusion of test compounds (endiandrin A [22], LPS, nintedanib, pioglitazone, or trametinib) or media-diluent control (media + DMSO). Prior to culture monocytes constituted on average 8.47% of the total cells (Supplemental Figure S1). After 72 hrs of culture the cells were harvested for analysis. In a pilot experiment we compared gene expression by qPCR after either lysing cells in situ on the plate, or trypsinising the cells, removing them from the plate and then extracting RNA. No significant differences were observed in the reference gene Ct values between compound treatment types (suggesting cell numbers at the time of harvest were similar between the groups), or in relative gene expression; suggesting that neither the recovery method nor the compound treatment were toxic and potentially skewing the scRNA-seq data (Supplemental Figure S2). For scRNA-seq experiments the cells were harvested by trypsinisation and fixed on the day of harvest then prepared for sequencing (material and methods). For scRNA-seq analysis, cells with less than 500 features (genes), or less than 1000 counts, or more than 20,000 counts (unique reads), or more than 5% mitochondrial counts were excluded (Supplemental Figure S3).

**Figure 1:**
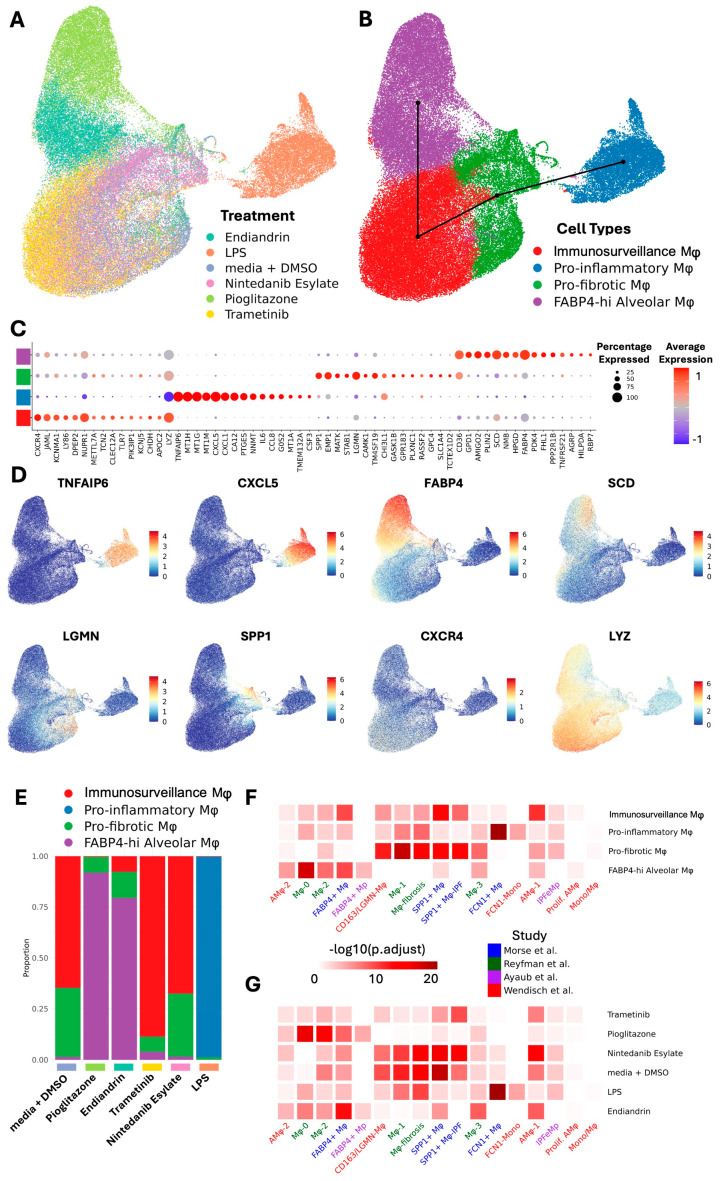
scRNA−seq reveals AM plasticity in response to treatment. AM scRNA-seq transcriptional profile. (**A**) UMAP embedding of AM scRNA-seq dataset coloured by treatment. (**B**) UMAP embedding of AM scRNA-seq dataset coloured by cell types overlayed with Slingshot trajectory analysis. (**C**) Dotplot showing expression of top 15 marker genes for each cell type in BAL. Dot size shows the percentage of cells with any mRNA counts and colour shows the scaled average expression of each gene across the cell type. (**D**) UMAP embedding of BAL scRNA-seq dataset showing mRNA molecule counts for different marker genes for different cell types. (**E**) Cell type proportion variation across treatments. (**F**) Heatmap representing −log10 transformed adjusted *p* values (one-sided Fisher’s exact test) assessing the overlap of gene sets (cluster markers) from alveolar macrophage subtypes to other published IPF BAL scRNA-seq macrophage/monocyte clusters. (**G**) Heatmap representing -log10 transformed adjusted *p* values (one-sided Fisher’s exact test) assessing the overlap of gene sets from alveolar macrophage groups modulated by different treatments to other published IPF BAL scRNA-seq macrophage/monocyte clusters.

Exposure to different compounds uniformly and differentially altered the transcriptome of the macrophages, suggesting a high level of plasticity (Figure 1A). Our analysis revealed four transcriptionally different macrophage populations we have termed FABP4^hi^, pro-inflammatory, pro-fibrotic, and immunosurveillance. Slingshot trajectory analysis [23] suggests a baseline in these cells within the immunosurveillance and pro-fibrotic subsets, with the FABP4^hi^ and pro-inflammatory subsets at opposite ends of a transcriptomic spectrum (Figure 1B). We can infer a logical function for these subsets from their signature genes (Figure 1C,D, and Supplemental Table S1). FABP4^hi^ macrophages are characterised by genes for lipid handling and metabolism (*FABP4* fatty acid binding protein 4, *SCD* fatty acid desaturase, *CD36* fatty acid translocase, *GPD1* Glycerol-3-phosphate dehydrogenase 1, *PLIN2* adipose differentiation-related protein). Pro-fibrotic macrophages are characterised by fibrosis-associated genes (*SPP1*, *LGMN*, *TM4SF19*, *CH3L1*). Inflammatory macrophages are characterised by a gene expression-profile associated with inflammation and LPS-stimulation (*LYZ* downregulation [lysozyme [24]), *TNFAIP6* [tumor necrosis factor-inducible gene 6 protein]; *CXCL5* & *CXCL1* [chemokine (C-X-C motif) ligand 5 & 1], *MT1H*, *MT1G* & *MT1M* [isoforms of Metallothionein 1]). Immunosurveillance macrophages are characterised by expression of genes for pathogen recognition (*TLR7*, *LYZ*, *LY86*, *CLEC12A*) and immune activation (*CXCR4*).

Pioglitazone strongly shifted the transcriptome towards the FABP4^hi^ profile (Figure 1E), and this is consistent with our predictions and observations by qPCR in our previous study [20] and in our pilot data (Supplemental Figure S2); and also predicted by others [13]. Similarly, the natural product endiandrin A shifted the transcriptome towards the FABP4^hi^ profile, although not as strongly as pioglitazone which is also consistent with our qPCR data. LPS strongly shifted the transcriptome towards the pro-inflammatory profile. Trametinib and nintedanib both tended to shift the transcriptome with uniformity specific to the compound, however, unlike the other compounds they did not drive the transcriptome into one clear subset, tending to fall in the immunosurveillance and pro-fibrotic profiles and this likely reflects that the compounds are only weakly active on AM as reflected by their low fold increases in expression of affected genes (Supplemental Figure S4 and Supplemental Table S2).

As mentioned earlier, there is consensus that AM share transcriptional profiles across different diseases [11,12,13,14,15] and this has important implications for therapeutic development, suggesting that driving transcription away from the pro-fibrotic program to the homeostatic program would be beneficial across different fibrotic conditions. To put our study into the context of the consensus data we assessed the transcriptional profiles in three published IPF datasets [11,12,13] and one post COVID-19 dataset [14]. Analysis of the overlap of gene set expression was performed (Figure 1F). The results suggest a strong similarity between our subsets and those of others, where: our homeostatic FABP4^hi^ AM population aligns with FABP4+, AMϕ-2, Mϕ-0, and Mϕ2 populations; our pro-fibrotic population aligns with the CD163/LGMN-Mϕ, Mϕ-1, Mϕ-fibrosis, SPP1+Mϕ, and SPP1+Mϕ-IPF populations. Importantly, treatment with pioglitazone or endiandrin A strongly altered transcription to resemble the consensus homeostatic populations (Figure 1G), supporting the idea that AM across different conditions are amenable to therapeutic transcriptomic reprogramming.

### 2.2. AM Transcriptional Reprogramming Occurs at the Transcriptional Level, Is Predictable, and Allows a Change in Functionality

**Figure 2:**
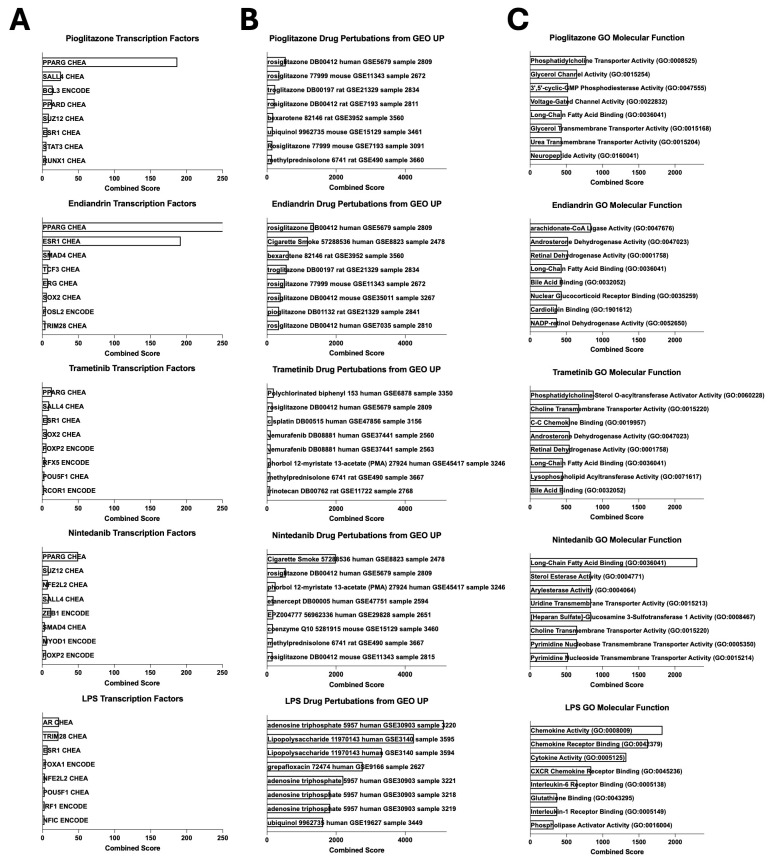
AM transcriptional reprogramming occurs at the transcriptional level, is predictable, and allows a change in functionality. The thirty most highly differentially expressed genes from each treatment group were assessed using Enrichr analysis (Enrichr generated Combined Score defined as natural log of the *p*-value from Fisher’s Exact Test multiplied by Z score of the deviation from the expected rank [25]). (**A**) Transcription factor analysis using ENCODE and ChEA Consensus TFs from ChIP-X dataset. (**B**) Drug perturbations using GEO up dataset (gene sets extracted from GEO comparing cells before and after treatment). (**C**) Molecular function using GO Molecular Function 2023 dataset.

We performed further analysis of the transcriptional profiles using the Enrichr gene set enrichment analysis tools [25,26,27]. Pioglitazone and endiandrin A treated cells both had a strong signal for the transcription factor PPARG which is not surprising as FABP4 expression is known to be driven by this transcription factor [28] (Figure 2A). A weaker PPARG signal was also seen in the trametinib and nintedanib samples and differentially increased PPARG expression is evident in the scRNA-seq data for all these treatment groups (except nintedanib) (Table S2). In contrast, the LPS response displayed a weak sex hormone transcription factor signal (androgen receptor AR) and TRIM28 signal, both known to be involved in LPS-induced responses [29,30]. We next asked if the transcriptional changes could be predicted or what drug perturbations were likely to induce the transcriptional profiles observed following the compound treatments: pioglitazone and endiandrin A were both dominated by a signal for glitazones, whilst LPS suggested adenosine triphosphate (ATP, a known extra-cellular pro-inflammatory molecule [31]) and LPS (Figure 2B). Finally, we asked if the function of the AM could be altered. Analysis of gene ontology molecular function indicated that the transcriptional profiles of all compound-treated subsets were dominated by lipid metabolism, except for LPS which drove chemokine and cytokine function (Figure 2C).

### 2.3. Plasticity in AM Transcriptomes Is Evident at the Chromosome Level

**Figure 3:**
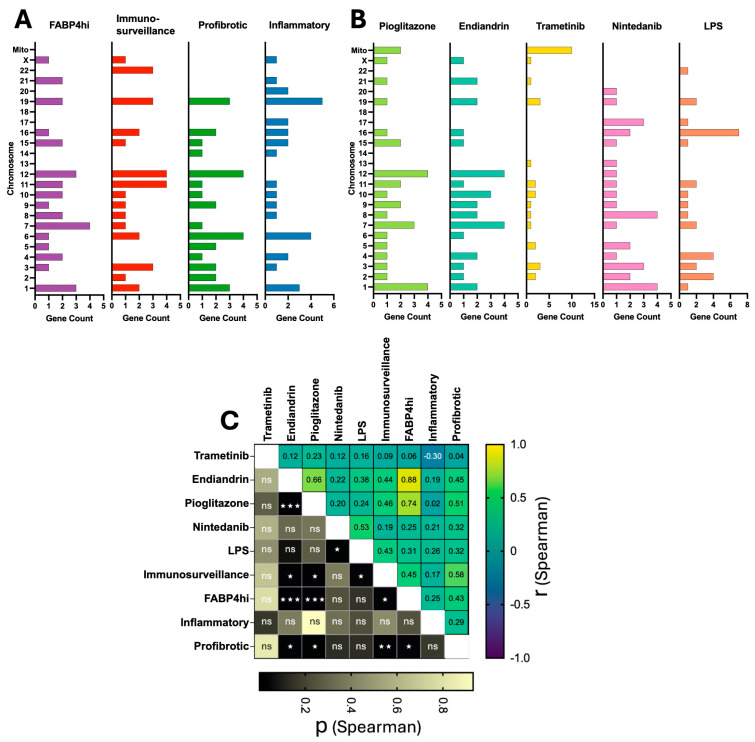
AM plasticity is evident at the chromosomal level. We assessed the chromosome location of the thirty most highly differentially expressed genes from our defined AM subsets (**A**) and from each treatment type (**B**). Correlation of chromosomal usage between cell subsets and treatment types (**C**) was assessed (Spearman correlation, two-tailed, GraphPad Prism 10.4.1, Spearman correlation coefficient (r, difference from zero) shown on *X* axis; and *p* value on *Y* axis (ns *p* > 0.05, ☆ *p* ≤ 0.05, ☆☆ *p* ≤ 0.01, ☆☆☆ *p* ≤ 0.001) (Mito = Mitochondria).

Next, we asked whether there was a bias in chromosomal usage in the different cell subsets, or following treatment (Figure 3A,B). We used a correlation analysis of the chromosomal location of the top 30 upregulated genes for each subset and treatment type. Within the cell types, the correlation tended to vary across the spectrum of the slingshot analysis (Figure 1B above) where the FABP4^hi^ subset had a higher correlation with the immunosurveillance and profibrotic groups than with the Inflammatory group (r spearman (r_s_) = 0.45, 0.43 and 0.25 respectively) (Figure 3C). Notably, chromosomal usage following treatment with pioglitazone or endiandrin A showed a moderately high correlation (r_s_ = 0.66) and a high correlation with the FABP4^hi^ subset (r_s_ = 0.74 and 0.88 respectively) all which were highly significant (*p* < 0.001). In contrast, treatment with trametinib induced a bias on mitochondrial genes including those for subunits of NADH dehydrogenase, cytochrome c oxidase, and ATP synthase: all of which are critical for oxidative phosphorylation (OXPHOS) [32], and although it is known that trametinib can increase OXPHOS [33], we believe this to be the first observation that indicates it acts by directly increasing mitochondrial gene expression of the OXPHOS pathway.

### 2.4. AM Transcriptional Plasticity Remains Following Drug Perturbation

**Figure 4:**
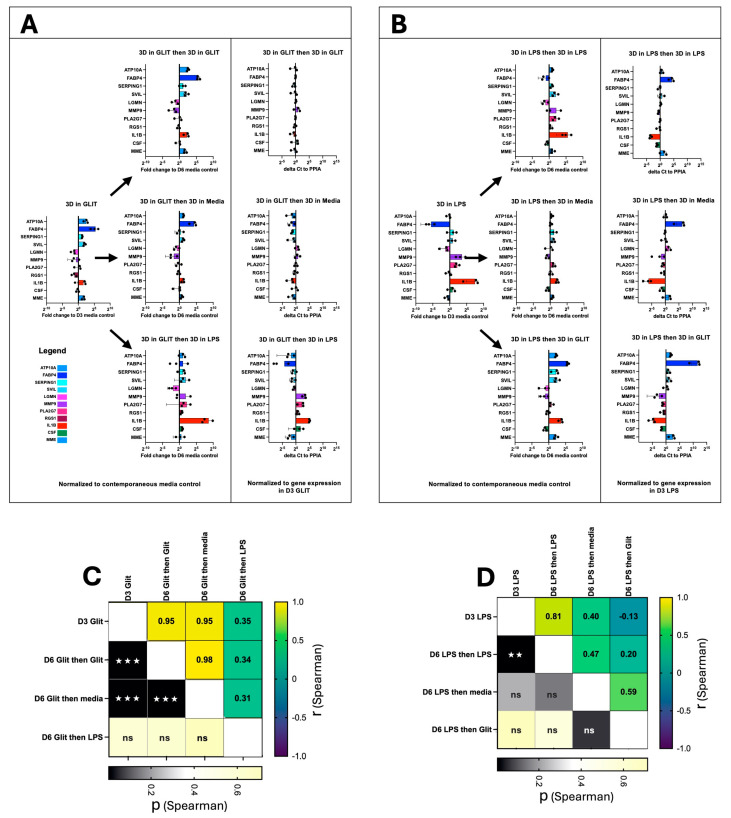
AM plasticity remains following perturbation. AM were cultured according to our standard procedure with the addition of either (**A**) Pioglitazone (Glit) or (**B**) LPS for 3 days at which point some cells were harvested and gene expression (as indicated) assessed by qPCR. The remaining wells were washed and re-cultured with the addition of either their original perturbagen, media only, or the alternate perturbagen. After 3 days of re-culture (Day 6) all cells were harvested and assessed by qPCR. At both timepoints the gene expression was compared to a control that had been in media only since Day 0 (n = 3, mean and SD shown). Similarity to original culture condition for (**C**) Pioglitazone or (**D**) LPS was assessed (Spearman correlation, two-tailed, GraphPad Prism, ns *p* > 0.05, ☆☆ *p* ≤ 0.01, ☆☆☆ *p* ≤ 0.001).

Finally, we asked whether the plasticity of AM remained after the initial compound treatment. AM were cultured for 3 days with exposure to either pioglitazone or LPS using our standard procedure (as above). The cells were then washed and re-cultured with either their original compound, media alone, or the alternate compound (Figure 4A,B). Gene expression of a select panel of genes was assessed by qPCR at day 3 (following the initial treatment), or at day 6 following the second culture. For cells initially cultured in pioglitazone, secondary culture in pioglitazone or media maintained the original gene expression profile (r_s_ = 0.95 and *p* < 0.001 in both cases); however, the expression profile no longer correlated with the original condition following secondary culture in LPS (r_s_ = 0.35, *p* = 0.3). A similar effect was observed for cells initially cultured in LPS: secondary culture in LPS maintained the expression profile (r_s_ = 0.81, *p* = < 0.01); however, the expression profile no longer correlated with the original condition following secondary culture in media or pioglitazone (r_s_ = 0.40, *p* = 0.22; and r_s_ = −0.13, *p* = 0.71 respectively).

## 3. Discussion

Herein we show that human AM possess a remarkable transcriptional plasticity and that they are readily amenable to pharmacological reprogramming. Using scRNA-seq following in vitro culture of AM exposed to different compounds we found that the entire population of AM were transcriptionally plastic and could be reprogrammed into distinct subsets that recapitulate different functional subsets observed in a growing body of ex-vivo work in humans and mice [9,10,11,12,13,14,15]. This observation is significant as there is consensus within that work that lung fibrosis is driven by an aberrant population of profibrotic AM and our work herein (and earlier [20]) indicate that these cells could be reprogrammed in an effort to halt fibrosis.

AM are unique cells with diverse roles. They reside in the airspace of the lung outside of the body, bathed in lung surfactant and constantly exposed to the external environment. They serve at least three major functions: (1) surfactant homeostasis and recycling; (2) immune surveillance and response (including initiating and driving inflammation) and (3) resolution of lung injury including driving tissue fibrosis and repair.

It is clear from the foregoing that AM need to be dynamic and perform sometimes opposing functions, so how is this achieved? Firstly, it is known that lung macrophages are potentially long-lived, they can self-renew, but they can also be replaced/renewed by monocytes that migrate to the lung air space from the blood and differentiate into cells that are eventually indistinguishable from other lung macrophages [34]. The last process likely takes some time, and it is worth noting that metabolic requirements of monocytes and macrophages are different, in the lung glucose levels are low (presumably to discourage bacterial growth), and lung macrophages are adapted to metabolise lipids [35], yet monocytes metabolise glucose so the differentiation process is complex and monocytes are not an instant replacement for macrophages. So the first model for dealing with the diverse requirements of lung macrophages is that distinct subsets of cells with fixed characteristics exist simultaneously; and this idea is supported at first glance by traditional methods and paradigms that have experimentally generated M1 or M2 macrophages or by newer scRNA-seq data that readily subsets macrophages into distinct sub-populations; however it is worth noting that these analyses are a snapshot in time and do not actually support the notion that the cell functions are fixed. The second model (supported by our work herein) is that the lung macrophages are transcriptionally plastic and can change to perform different roles as required. From a therapeutic viewpoint the idea that lung macrophages are transcriptionally plastic is appealing because it suggests that they may be readily amenable to pharmacological re-programming and, for example, changing them from being aberrantly pro-fibrotic to homeostatic.

Here we found that treatment of AM with pioglitazone or the natural compound endiandrin A induced a transcriptional reprogramming to match the homeostatic profile of the consensus FABP4^hi^ subset and its homologues (Figure 1F) that appears to act via the transcription factor PPARG. In a previous study [20], we predicted that glitazones would have this effect based on analysis of published scRNA-seq data and interrogation of readily accessible online gene set enrichment analysis databases such as Enrichr [25,26,27]. Moreover, re-interrogating drug perturbation libraries with our transcriptional data generated herein with pioglitazone, we again find that glitazones are the top perturbagens (Figure 2B); and in contrast, treatment with LPS induced an inflammatory transcriptional profile that could also be predicted (Figure 2B). These observations are important as they validate the use of these tools in aiding drug discovery.

We also assessed chromosomal bias in the transcriptional profile of the different cell subsets and following the different compound treatments. Chromosomal bias tended to follow the slingshot trajectory analysis, further supporting the notion that the inflammatory and FABP4^hi^ subset sit at each end of a transcriptional and functional spectrum. Furthermore, the high correlation observed between the pioglitazone, endiandrin A, and FABP4^hi^ subset is interesting because it suggests that standard transcriptional programs exist in AM to drive the cells towards a certain function. So, two very different compounds (pioglitazone and endiandrin A) can have a similar effect on the AM, perhaps suggesting that transcriptional plasticity operates somewhat linearly, consistent with the slingshot analysis. The mitochondrial bias observed in the trametinib-treated cells was unexpected and is particularly interesting. Firstly we do not believe this to be an aberration or an indicator of cell death as our quality controls and all indicators (cell viability and qPCR reference gene Ct values) are acceptable, and as mentioned earlier trametinib is known to upregulate OXPHOS [33], however this direct effect on OXPHOS genes was unknown. So this observation is important as it gives another dimension to the plasticity of the AM, but also highlights an underrecognized bioinformatic oversight where some gene set enrichment analysis do not include mitochondrial genes and the arbitrarily-set mitochondrial gene cut-off in scRNA-seq may need modifying [36].

Finally, we asked whether AM retain their plasticity after initial drug treatment: AM initially treated with either pioglitazone or LPS tended to maintain their gene expression profile when re-cultured in the same compound or even in media alone. This suggests that the initial stimulus induces a relatively stable change in gene expression that persists even after the removal of the stimulus (in the case of media alone). However, the plasticity is evident when the AM are switched to the alternate compound. Cells initially treated with pioglitazone lose their pioglitazone-specific expression profile when switched to LPS, and vice versa. This indicates that the second stimulus overrides the memory of the first, driving the cells towards a new transcriptional state characteristic of the second compound. It is interesting to note that day 3 LPS-treated cells, restimulated with LPS continued to express pro-inflammatory cytokines at day 6, albeit at a reduced rate when compared to day 3. A large body of work suggests that LPS treatment of monocytes and macrophages tolerizes them against further LPS stimulation (reviewed in [37]); however, human alveolar macrophages appear different in this respect and can continue to respond to LPS treatment by producing pro-inflammatory cytokines (reviewed in [38]). Furthermore, a large body of data has suggested that macrophages can polarise into two main states, M1 (pro-inflammatory), or M2 (anti-inflammatory) ([39]) and that M1 macrophages are resistant to reprogramming to M2 ([40]); however, much of this data is generated with monocyte derived macrophages from humans and mice and those rigid classifications do not apply to human alveolar macrophages as demonstrated by our study herein, and the numerous scRNA-seq studies of human alveolar macrophages ([9,10,11,12,13,14,15]).

We acknowledge some limitations in our study. Firstly, we utilise in vitro culture of human alveolar macrophages using relatively standard cell culture methods including the presence of glucose and the absence of lung surfactant which present a different milieu to the native lung. Secondly, the alveolar macrophages used in our study are from patients diagnosed with silicosis and are not “naïve”. Nevertheless, we believe these factors, while worth noting, do not negatively impact our observations and conclusions.

Taken together, the work presented herein provides strong evidence that human AM are transcriptionally plastic thereby allowing them to modify their function as required to maintain homeostasis, whilst also being able to drive inflammation and fibrosis when required. Importantly, these findings strongly support the notion that profibrotic macrophages are amenable to pharmacological reprogramming and that we have the existing tools to identify putative drugs. The challenge moving forward is to establish in vivo models that accurately recapitulate human disease and cell phenotypes so we can bridge the final gap to the clinic.

## 4. Materials and Methods

### 4.1. Collection and Cryopreservation of Human AM

As described earlier [20], briefly, whole lung lavage washout, collected from patients participating in a silicosis treatment trial at our hospital [21], was processed to isolate and cryopreserve AM. The lavage fluid, collected in 3-litre containers, was transported to our lab, processed and centrifuged. The resulting cell pellet was resuspended in R2 media (RPMI 1640 medium supplemented with 2% FCS and penicillin/streptomycin/glutamine (all Gibco, Thermo Fisher Scientific, Seventeen Mile Rocks, Australia) and then mixed with an equal volume of FCS containing 15% DMSO (Sigma, Merck Life Science, Bayswater, Australia). This cell suspension was then divided into 40 × 1 mL aliquots and frozen in liquid nitrogen for later use.

### 4.2. Cell Culture Conditions

Cells from one patient (patient ID 236) were thawed and washed in R2 media. Live cells were then counted in a haemocytometer using trypan blue stain (Gibco, Woolloongabba, Australia), resuspended in Dulbecco’s modified Eagle’s medium (DMEM) (Gibco) supplemented with 10% FCS (Gibco), 0.01M Hepes solution (Sigma, Merck Life Science, Bayswater, Australia) and penicillin/streptomycin/glutamine (Gibco) (complete DMEM) and 1.2 × 10^6^ cells per well were added to 12-well plates (Costar 3513, Corning, Merck, Bayswater, Australia) in 2.5 mL per well, inner wells only, with one well per scRNA-Seq sample. Outer wells in each plate were left cell-free and 2 mL of PBS (Gibco) was added. After incubating cultured cells overnight at 37 °C in 5% CO_2_, the media was aspirated from the cells and replaced immediately with 1.5 mL complete DMEM. A further 1 mL was added per well of either media containing the compound of interest or DMSO as the negative control, at the concentration required to achieve the desired final concentration. Compounds added were pioglitazone-HCl (Sandoz, North Sydney, Australia) 10 µM, endiandrin A [22,41] (Davis Open Access Natural Product Library, Institute for Biomedicine and Glycomics, Griffith University) 20 µM, trametinib (MedChemExpress, Monmouth Junction, NJ, USA; Catalogue Number HY-L022) 10 µM, nintedanib esylate (MedChemExpress; Catalogue Number HY-L022) 10 µM, LPS (InvivoGen, San Diego, CA, USA) 100 ng/mL or DMSO (Sigma) 1 µL/mL. All other wells were incubated at 37 °C in 5% CO_2_ for a further 72 h (day 4) before harvesting each well for scRNA-seq sample preparation.

### 4.3. Preparation of Cells for Performing scRNA-Seq

All samples were prepared ready for scRNA-Seq analysis on the day of harvest from their culture well. Cells were harvested by aspirating off the media, then the adherent cells were washed with 2 mL PBS and incubated with 750 µL Trypsin-EDTA (Gibco) at 37 °C in 5% CO_2_ for ~5–6 min. Cells were then removed and washed in R2 media and then centrifuged 400× *g*, 5 min, 4 °C and resuspended in 1 mL PBS with 0.04% FCS to wash twice, then 70 µM filtered. Live cells were counted in a haemocytometer using trypan blue stain, then centrifuged and resuspended in 1 mL of freshly prepared fixation buffer, containing 1× concentration of 10× concentrated Fix & Perm Buffer (10x Genomics PN-2000517) and 4% formaldehyde, and incubated overnight at 4 °C. Cells were centrifuged at 850× *g* for 5 min then the fixation buffer was aspirated off and cells were resuspended and mixed gently with 1 mL of chilled 1× concentration of 8× concentrated quenching buffer (10x Genomics PN-2000516). 100 µL of Enhancer (10x Genomics PN-2000482), pre-warmed at 65 °C for 10 min, was added and mixed with a pipette, then 275 µL of 50% glycerol was added for a final concentration of 10%, pipette mixed, and all samples were stored at −80 °C until scRNA-seq was performed.

### 4.4. scRNA-Seq

Fixed cells (as above) were processed using the 10x Genomics Chromium Fixed RNA Profiling (FLEX) kit, following the manufacturer’s protocol (CG000527, 10x Genomics). Single-cell gel bead emulsions (GEMs) were generated using the Chromium X System (10x Genomics). The final libraries were sequenced on an Illumina NextSeq 2000 (Illumina, Inc.; San Diego, CA, USA) using a dual-indexed sequencing configuration: Read1: 28 cycles, i7 index: 10 cycles, i5 index: 10 cycles, Read2: 90 cycles.

### 4.5. scRNA-Seq Bioinformatic Analysis

AM scRNA-seq count matrices for each sample were loaded into Seurat 5.0.1 [42] and cells with less than 500 features or less than 1000 counts or more than 20,000 counts or more than 5% mitochondrial counts were excluded. Samples were then merged and processed using Seurat functions SCTransform, RunPCA, FindNeighbors (using 20 PCs), RunUMAP, FindClusters (resolution = 0.1). Marker genes for each cluster were identified using FindAllMarkers and these were used to annotate clusters. Trajectory analysis was run using Slingshot 2.10.0. [23]. For assessing marker gene overlap with clusters from other studies, a one-sided Fisher’s exact test for overlap was run and *p* values were corrected using the Benjamini-Hochberg procedure.

### 4.6. Cell Culture for Experiments Assessing Plasticity Following Serial Compound Treatment

Cells from 3 patients (patient IDs 119, 158 and 236) were thawed, counted and 7.5 × 10^5^ cells were cultured as described above, 1.5 mL per well in media (complete DMEM) alone in 24-well plates (Costar 3526) and incubated at 37 °C in 5% CO_2_ for 24 h. The media was aspirated from the wells and immediately replaced with media containing pioglitazone 10 µM, LPS 50 ng/mL or DMSO 1 µL/mL and incubated at 37 °C in 5% CO_2_ for 72 h. Cells were then harvested using trypsin-EDTA as described, counted, and each divided 4 ways for an RNA sample resuspended in 300 µL of RLT lysis buffer (Qiagen RNeasy micro kit) and stored at −20 °C for later RNA extraction, or re-cultured and incubated for a further 72 h with pioglitazone 10 µM, LPS 50 ng/mL or DMSO 1 µL/mL in 150 µL per well in 96-well flat-bottomed plate (Costar 3596). Media was removed by transferring into a 96-V-well plates (Costar 3897) and immediately replaced with 150 µL of RLT lysis buffer onto the adherent cells, mixing well. Loose cells in the media were centrifuged in the V-well plate, media aspirated away and the RLT containing the lysed adherent cells was transferred to the cell pellets and mixed. The plates were stored at −20 °C until RNA was extracted using the protocol supplied with the Qiagen RNeasy micro kit. Cultures were viewed and photographed microscopically at all time points prior to harvesting.

### 4.7. Quantitative PCR Analysis

All extracted RNA was transferred to 96-well PCR plates (Applied Biosystems, Thermo Fisher Scientific, Seventeen Mile Rocks, Australia) and cDNA synthesis was performed using iScript^TM^ Select cDNA Synthesis kit (Bio-Rad Laboratories, Inc., Hercules, CA, USA). Single-plex qPCR was then performed in 384-well PCR plates (Applied Biosystems) in a Quant Studio 5 PCR machine (Applied Biosystems) using SsoAdvanced^TM^ Universal Probes Supermix (Bio-Rad) and Taqman Gene Expression kits (Applied Biosystems) as described earlier [20]. Test genes were normalised to PPIA expression using the delta Ct method. All qPCR presented herein includes data from 3 patients (IDs 119, 158 and 236).

### 4.8. Chromosome Location

Chromosomal location of differentially expressed genes was assessed and downloaded using the multi-symbol checker function on the HGNC website [43]. Spearman correlation was performed using GraphPad Prism Version 10.4.1.

## Data Availability

The data that support the findings of this study are available on request from the corresponding author. The data are not publicly available due to privacy or ethical restrictions.

## Supplementary Materials

The following supporting information can be downloaded at: https://www.mdpi.com/article/10.3390/ijms26094439/s1.
