# Supplementary figures and images for: Transcriptomic Plasticity of Human Alveolar Macrophages Revealed by Single-Cell RNA Sequencing Following Drug Exposure: Implications for Therapeutic Development
Source: Int J Mol Sci. 2025 May 7;26(9):4439. doi: 10.3390/ijms26094439 (PMC12072627; doi:10.3390/ijms26094439)

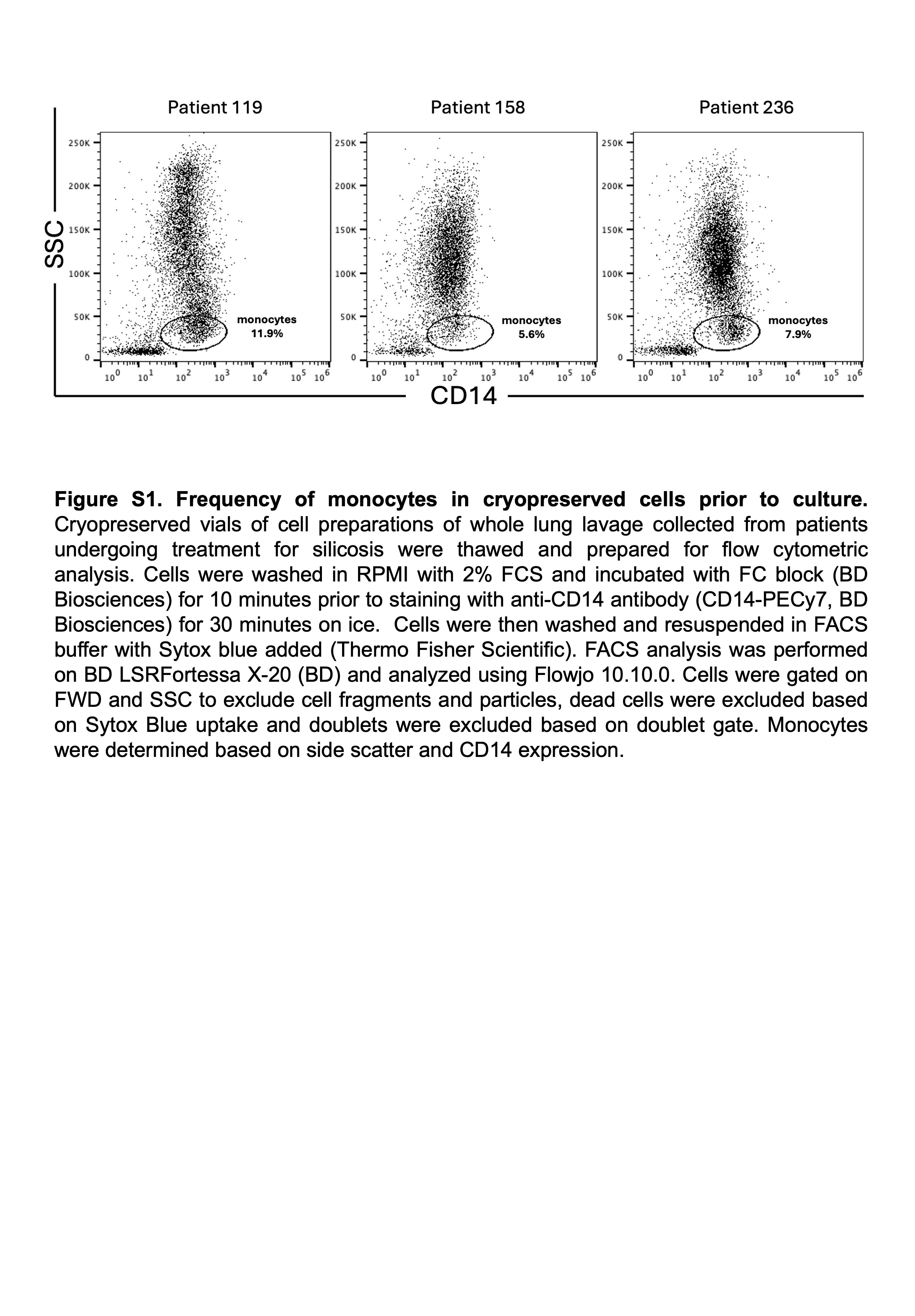

Supplement: Supplementary file 1 [file ijms-26-04439-s001.zip › Figure S1 with Legend.jpg]

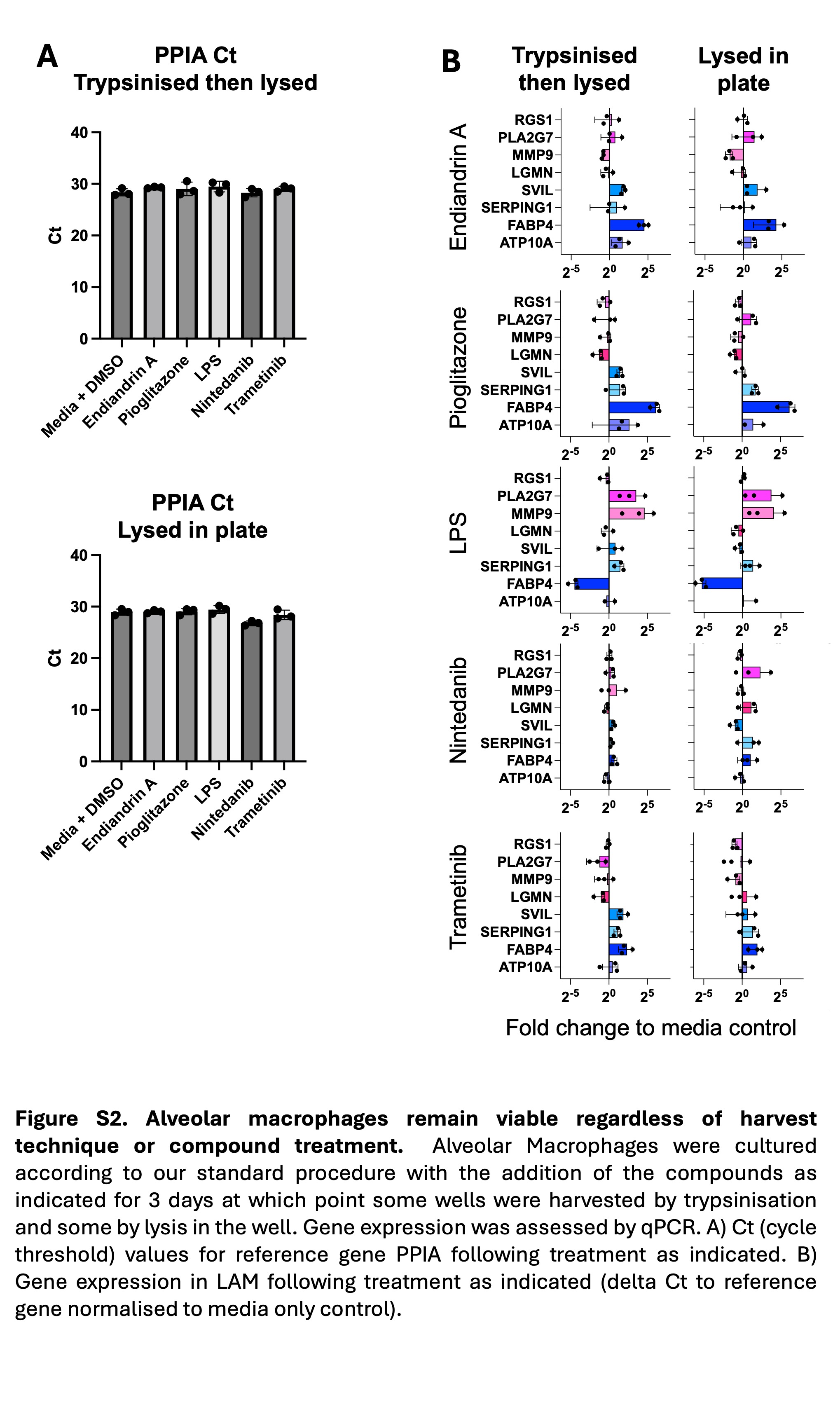

Supplement: Supplementary file 1 [file ijms-26-04439-s001.zip › Figure S2 with Legend.jpg]

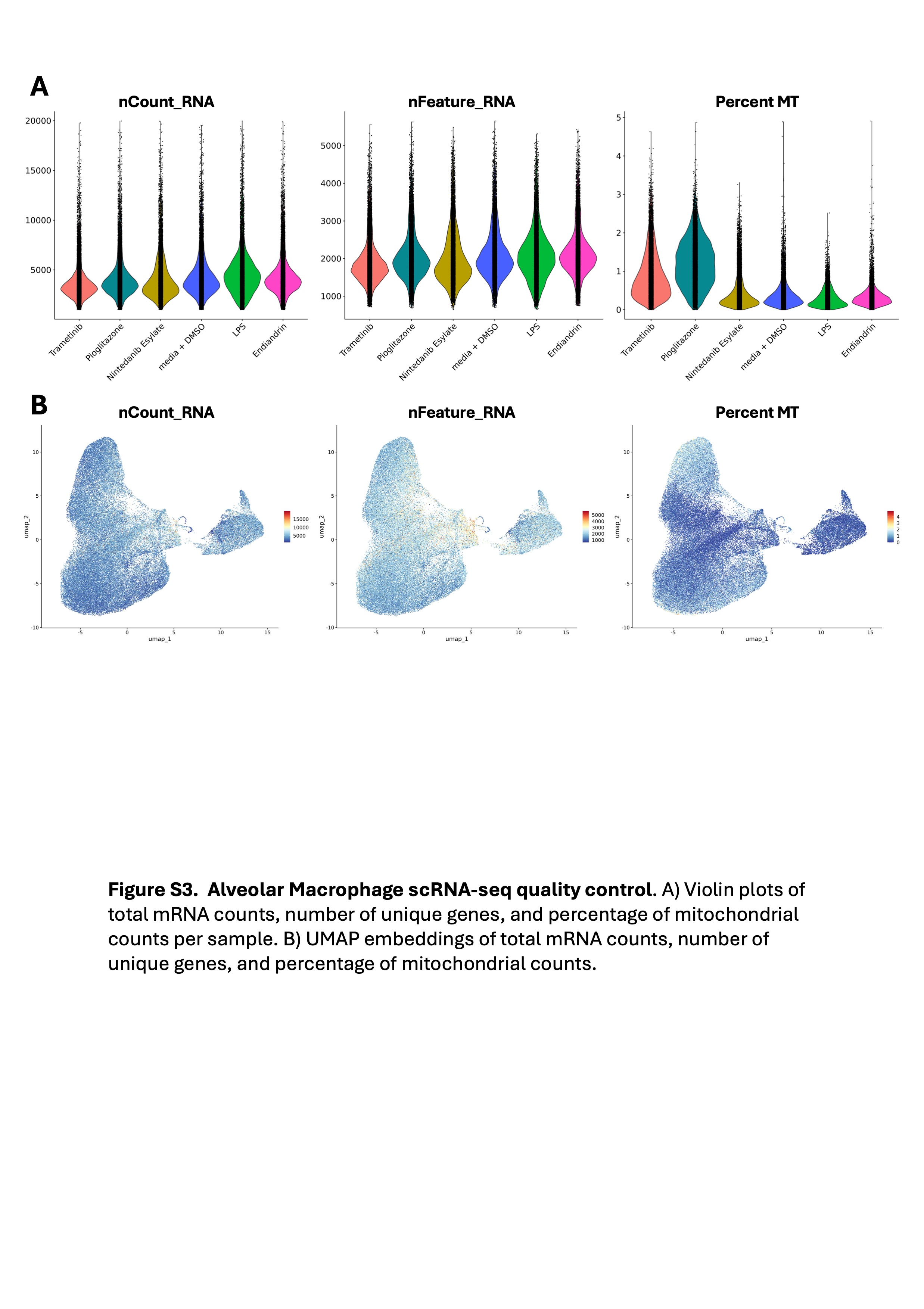

Supplement: Supplementary file 1 [file ijms-26-04439-s001.zip › Figure S3 with Legend.jpg]

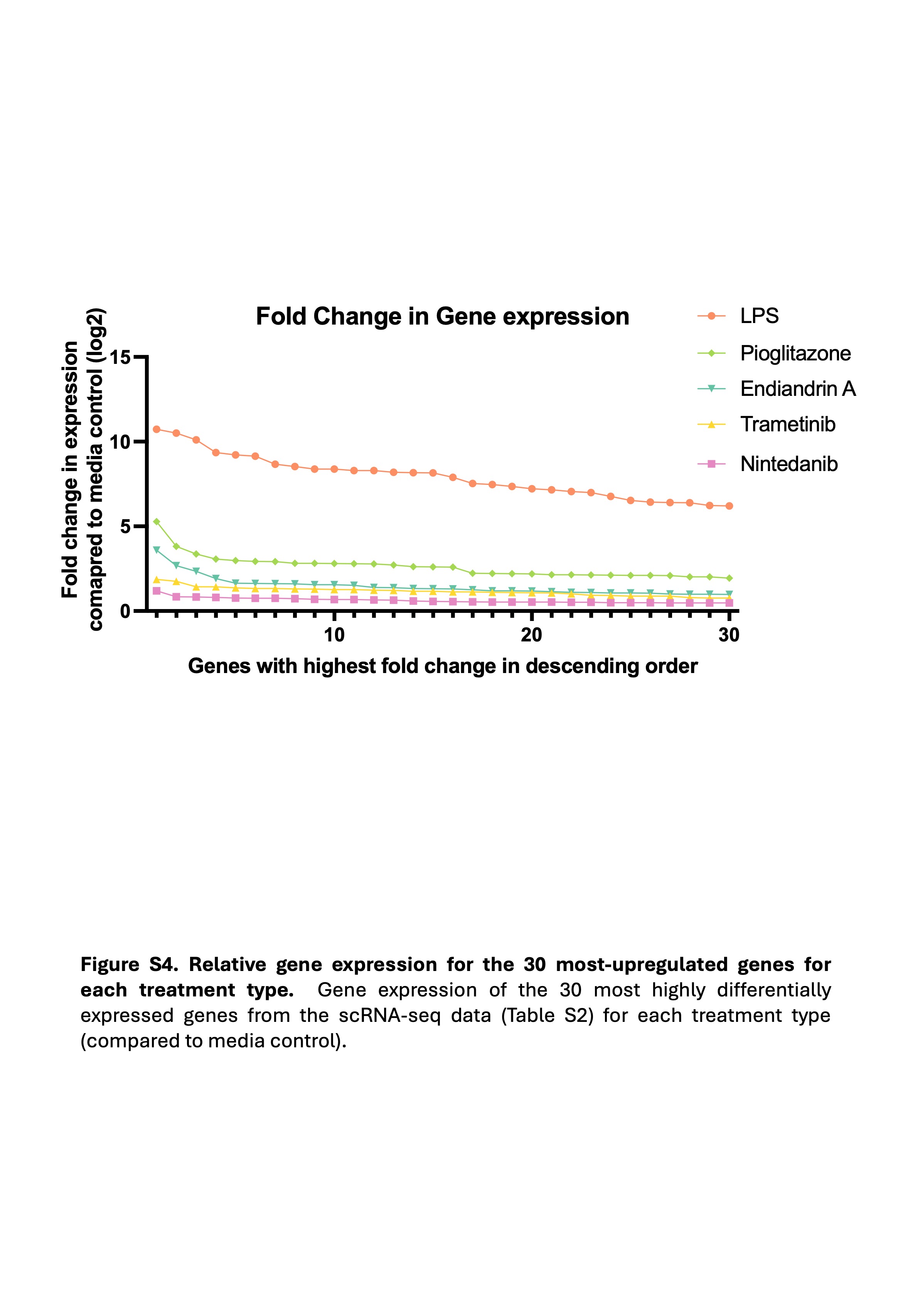

Supplement: Supplementary file 1 [file ijms-26-04439-s001.zip › Figure S4 with Legend.jpg]
